# Integrated Bacteria-Fungi Diversity Analysis Reveals the Gut Microbial Changes in Buffalo With Mastitis

**DOI:** 10.3389/fvets.2022.918541

**Published:** 2022-06-27

**Authors:** Xiushuang Chen, Miao An, Wenqian Zhang, Kun Li, Muhammad Fakhar-e-Alam Kulyar, Kun Duan, Hui Zhou, Yu Wu, Xin Wan, Jianlong Li, Lingtong Quan, Zhanhai Mai, Wenxia Bai, Yi Wu

**Affiliations:** ^1^College of Veterinary Medicine, Nanjing Agricultural University, Nanjing, China; ^2^MOE Joint International Research Laboratory of Animal Health and Food Safety, College of Veterinary Medicine, Nanjing Agricultural University, Nanjing, China; ^3^College of Veterinary Medicine, Huazhong Agricultural University, Wuhan, China; ^4^China Tobacco Henan Industrial Co. Ltd., Zhengzhou, China; ^5^College of Veterinary Medicine, Xinjiang Agricultural University, Urumqi, China; ^6^College of Life Sciences, Nanjing Agricultural University, Nanjing, China; ^7^Nanjing Superbiotech Co. Ltd., Nanjing, China

**Keywords:** gut microbiota, bacterial, fungal, buffalo, mastitis

## Abstract

The gut microbial community is closely related to mastitis, but studies regarding the influences of mastitis on gut microbiota in buffalo remain scarce. Herein, we characterized the differences in gut bacterial and fungal communities between mastitis-affected and healthy buffalos. Interestingly, although mastitis had no effect on gut bacterial and fungal diversities in the buffalos, some bacterial and fungal taxa were significantly altered. Bacterial and fungal taxonomic analysis showed that the preponderant bacterial phyla (Firmicutes and Bacteroidetes) and fungal phyla (Ascomycota and Basidiomycota) in buffalo were the same regardless of health status. At the level of genus, the changes in some gut bacterial and fungal abundances between both groups were gradually observed. Compared with healthy buffalos, the proportions of 3 bacterial genera (*uncultured_bacterium_f_Muribaculaceae, Eubacterium_nodatum_group*, and *Lachnoclostridium_10*) and 1 fungal genus (*Pichia*) in the mastitis-affected buffalo were significantly increased, whereas 4 bacterial genera (*Ruminococcus_2, Candidatus_Stoquefichus, Turicibacter*, and *Cellulosilyticum*) and 4 fungal genera (*Cladosporium, Thermothelomyces, Ganoderma* and *Aspergillus*) were significantly decreased. Taken together, this research revealed that there was significant difference in the compositions of the gut microbial community between the healthy and mastitis-affected buffalos. To our knowledge, this is the first insight into the characteristics of the gut microbiota in buffalos with mastitis, which is beneficial to understand the gut microbial information of buffalo in different health states and elucidate the pathogenesis of mastitis from the gut microbial perspective.

## Introduction

Mastitis, an inflammatory response of the mammary parenchyma, affects almost all lactating mammals especially high-yield cows (1). It can lead to decreased milk production, severely restraining dairy industry development (2). Early investigation revealed that fecal microbiota transplantation from cows with mastitis to germ-free mice caused inflammations in multiple tissues, such as colon, spleen, and serum, as well as mastitis symptoms in the mammary gland (3). Moreover, probiotic administration has been demonstrated to effectively alleviate mastitis symptoms in some exploratory human clinical trials, indicating that the mechanism of mastitis protection may be mediated through the gut microbiota (4).

Growing evidence indicated that gut microbiota participated in multiple physiological and metabolic functions of the host, including nutrient acquisition, intestinal epithelium differentiation, and intestinal metabolism (5–8). Moreover, the gut microbiota has also been demonstrated to play role in intestinal mucosal barrier and immune system maturation, implying its contribution in disease prevention and immunologic functions (9, 10). However, multiple environmental-related factors, such as diet, nutritional deficiencies, antibiotic treatment, and exposure to contaminants, may affect gut microbial homeostasis or even induce gut microbial dysbiosis (11, 12). Stable gut microbiota enabled the intestines to function properly, whereas gut microbial dysbiosis may cause etiopathologic consequences (13, 14). Currently, gut microbial dysbiosis has been shown to be the core and critical factor of many gastrointestinal diseases, such as colonitis and diarrhea (15, 16). Additionally, disturbed gut microbiota and its metabolites could pass through the intestinal mucosal barrier and affect peripheral organ systems by blood circulation, causing physiological dysfunction and even disease, such as lipid disorders, diabetes, and non-alcoholic fatty liver (17, 18).

Recently, culture-independent techniques, mainly including metagenomic and 16S rDNA amplicon sequencing, have been successfully developed and widely applied to dissect the complicated gut microbial ecosystem, as well as investigate gut microbial alterations after suffering certain diseases (19, 20). By systematically investigating and analyzing the microbial information acquired, we can further understand the gut microbiota-host interaction and mechanisms contributing to ill-health, thereby formulating effective measures to minimize the collateral damage. Presently, high-throughput sequencing technologies have successfully dissected the gut microbiota of giraffes, yaks, goats, and dairy cattle, making considerable contributions to the etiological analysis, diagnosis, and treatment of multiple gastrointestinal and systemic diseases (5, 21, 22). As an important source of protein acquisition for humans, buffalo milk has increasingly attracted widespread attention due to its high fat, protein, mineral, and vitamin contents. However, mastitis dramatically decreases buffalo milk production and quality, causing significant health and economic burden in buffalo farming. Although the gut microbial importance in host health is widely acknowledged, scarce knowledge is known about the interaction between mastitis and gut microbiota in buffalo. Herein, we investigated the gut bacterial and fungal shifts of buffalo with mastitis.

## Materials and Methods

### Animals and Sample Collection

In this investigation, 10 buffalos (5 healthy and 5 with mastitis) in Jingzhou, China were used for sample acquisition, and all selected buffalos had similar characteristics, including age, weight, diet, immune background, and dwelling environment. Buffalo mastitis was diagnosed by the California mastitis test (CMT) using a commercial kit. Moreover, the confirmed cases did not receive any treatment prior to the sample collection. On the day of sample collection, all the selected buffalos were placed in separate areas to maximally decrease potential contamination among different samples of subjects. The sterilized fecal samplers were used for collecting the rectal feces of each buffalo. The collected samples were immediately placed intosterile plastic containers and transported to the laboratory and later stored at −80°C for further study.

### 16S rDNA and ITS Genes Amplicon Sequencing

Five fecal samples (~200 mg) from healthy and mastitis-affected buffaloes were unfrozen and homogenized before DNA extraction. Subsequently, the bacterial and fungal DNA of the processed fecal samples were extracted using QIAamp DNA Mini Kit (QIAGEN, Hilden, Germany) based on the manufacturer's recommendations. The quality and quantity of the gDNA were evaluated *via* 0.8% (w/v) agarose gel electrophoresis and UV-Vis spectrophotometer (NanoDrop 2000, United States), respectively. To characterize the gut bacterial and fungal shifts, we amplified the V3/V4 and ITS2 regions utilizing bacterial (338F: ACTCCTACGGGAGGCAGCA and 806R: GGACTACHVGGGTWTCTAAT) and fungal (ITS5F: GGAAG TAAAAGTCGTAACAAGG and ITS2R: GCTGCGTTCTTCATCGA TGC) primers, respectively. PCR amplification was conducted as per the procedure previously described (5, 6). PCR products were subjected to target fragment recovery and quality evaluation and gel electrophoresis to obtain purified products. The recovered products were quantified by fluorescence using Quant-iT PicoGreen dsDNA Assay Kit on the Promega QuantiFluor fluorescent quantitative system and the libraries with a concentration above 2 nM and only one peak were considered qualified. The final purified products were applied for preparing the sequencing library using MiSeq Reagent Kit V3 (600 cycles) on the MiSeq sequencing machine.

### Bioinformatics and Statistical Analysis

The raw data was requested to be preprocessed. Specifically, quality detection and primer removal were applied to initial data with some problematic sequences, including unqualified, short, or mismatched sequences, to acquire clean reads through the Trimmomatic (v0.33) and Cutadapt software (1.9.1). The collected clean reads were subjected to splice and secondary filter as per the length range of different regions using Usearch software (v10). Afterward, recognition and removal of chimera sequences were conducted to achieve effective reads. The effective reads with 97 similarities were clustered into the same operational taxonomic unit (OTU). Moreover, Venn graphs were also generated to visualize the OTUs abundance and distribution in each group. To further dissect the influence of mastitis on gut microbial diversity and abundance, we computed multiple alpha diversity indexes according to the OTUs' distribution. On the other hand, beta diversity analysis was used for characterizing the differences between gut bacterial and fungal principal components. The assessment of sequencing depth for each sample was based on rank abundance and rarefaction curves. Differential bacterial and fungal taxa were determined by the LEfSe and Metastats analysis. Data analysis was performed by SPSS statistical program (v20.0) and *P*-values (means ± SD) <0.05 were recognized as statistically significant.

## Results

### Sequence Analysis

**Figure 1:**
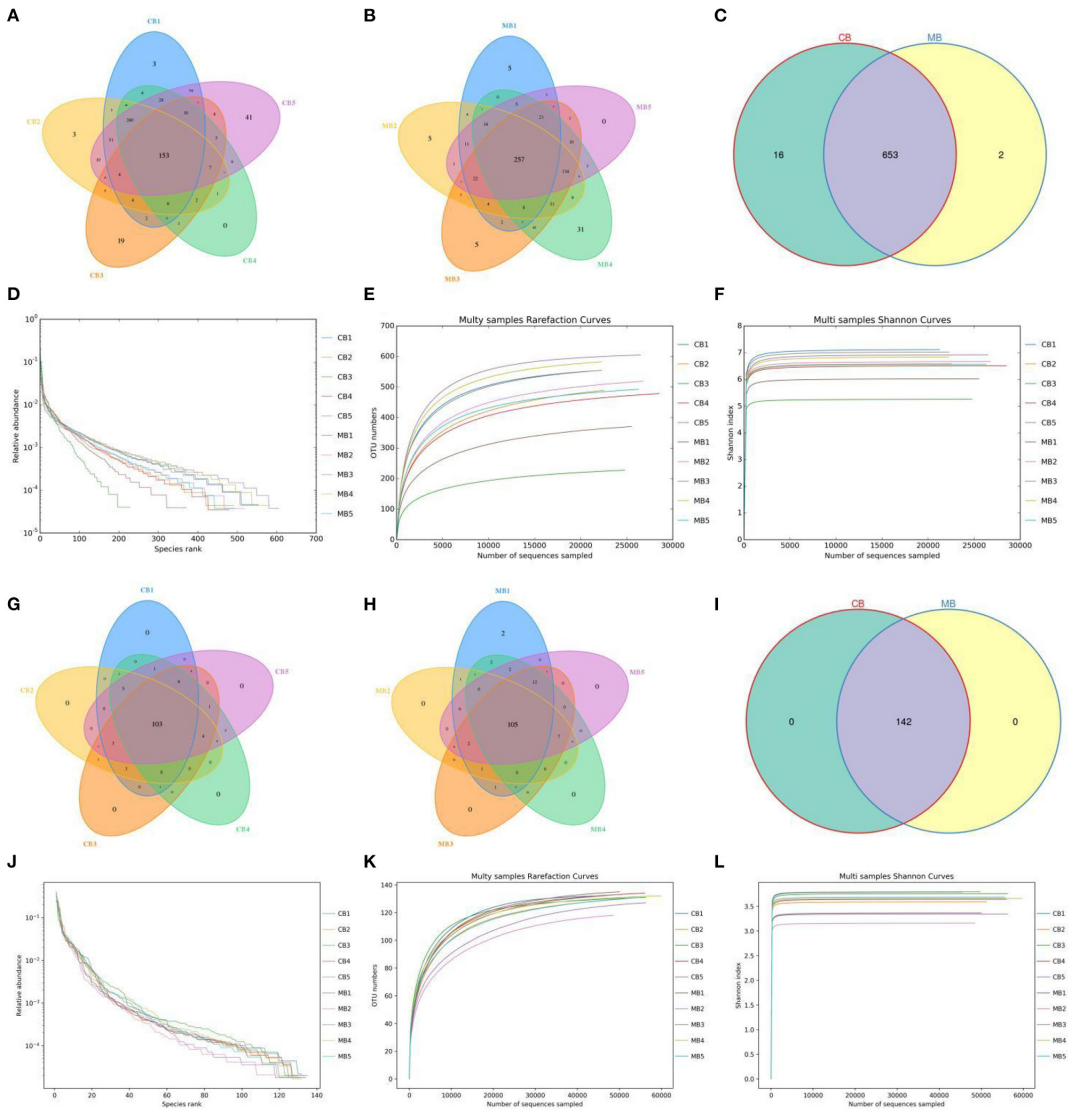
Operational taxonomic units (OTUs) distribution and sequencing data analysis. Venn diagrams for gut bacterial **(A–C)** and fungal **(G–I)** OTUs distribution. Gut bacterial **(D–F)** and fungal **(J–L)** sequencing depth and evenness were assessed by rank abundance and rarefaction curves.

In this study, we collected a total of 10 fecal samples for amplicon sequencing and 535,789 (CB = 264,271, MB = 271,518), and 707,929 (CB = 353,169, MB = 354,760) original sequences were achieved from the gut bacterial and fungal communities, respectively (Table 1). After quality assessment, 906,588 (CB = 372,157, MB = 534,431) eligible sequences were identified, with a median read count of 372,15 (ranging from 306,42 to 452,06) and 534,43 (ranging from 462,29 to 603,82) reads from bacterial V3/4 and fungal ITS2 regions from each sample, respectively (Table 2). The qualified sequences were clustered into 671 bacterial OTUs and 142 fungal OTUs as per 97% sequence similarity (Figures 1A–C,G–I). Additionally, the amounts of unique bacterial OTUs in CB and MB were 16 and 2 and 653 OTUs were shared in both groups, accounting for approximately 97.31% of the total bacterial OTUs. Meanwhile, we also observed 142 common OTUs in CB and MB, which consisted of more than 100% of the overall fungal OTUs. The results of accumulation and rarefaction curves demonstrated that almost all species can be detected (Figures 1D–F,J–L).

**Table 1:** Bacterial sequence information from amplicon sequencing.

**Sample**	**Raw reads**	**Clean reads**	**Effective reads**	**AvgLen (bp)**	**GC (%)**	**Q20 (%)**	**Q30 (%)**	**Effective (%)**
CB1	48,793	35,977	33,927	413	52.44	99.90	99.32	69.53
CB2	48,184	35,934	33,823	415	52.01	99.89	99.24	70.20
CB3	45,977	35,014	30,642	409	53.04	99.89	99.30	66.65
CB4	56,419	41,677	39,317	414	52.23	99.90	99.30	69.69
CB5	64,898	48,378	45,206	410	53.00	99.90	99.35	69.66
MB1	52,600	38,853	36,284	413	52.50	99.90	99.33	68.98
MB2	58,347	44,008	41,694	413	52.29	99.91	99.34	71.46
MB3	46,523	34,649	32,959	412	52.75	99.90	99.28	70.84
MB4	54,475	39,900	38,014	411	53.01	99.89	99.32	69.78
MB5	59,573	42,703	40,291	410	53.25	99.91	99.34	67.63

**Table 2:** Fungal sequence information from amplicon sequencing.

**Sample**	**Raw reads**	**Clean reads**	**Effective reads**	**AvgLen (bp)**	**GC (%)**	**Q20 (%)**	**Q30 (%)**	**Effective (%)**
CB1	63,677	46,541	46,229	211	46.99	99.98	99.92	72.60
CB2	74,384	52,096	51,858	191	41.95	99.98	99.94	69.72
CB3	71,417	57,065	56,948	172	37.96	99.99	99.95	79.74
CB4	70,972	56,774	56,609	179	38.77	99.99	99.95	79.76
CB5	72,719	56,777	56,667	166	34.39	99.99	99.97	77.93
MB1	69,180	50,752	50,668	168	36.58	99.98	99.95	73.24
MB2	71,736	48,949	48,859	163	34.15	99.97	99.93	68.11
MB3	70,243	50,350	50,268	179	36.95	99.98	99.95	71.56
MB4	72,266	60,497	60,382	180	36.45	99.99	99.96	83.56
MB5	71,335	56,069	55,943	166	35.48	99.99	99.95	78.42

### Microbial Diversities Analysis Associated With Mastitis

**Figure 2:**
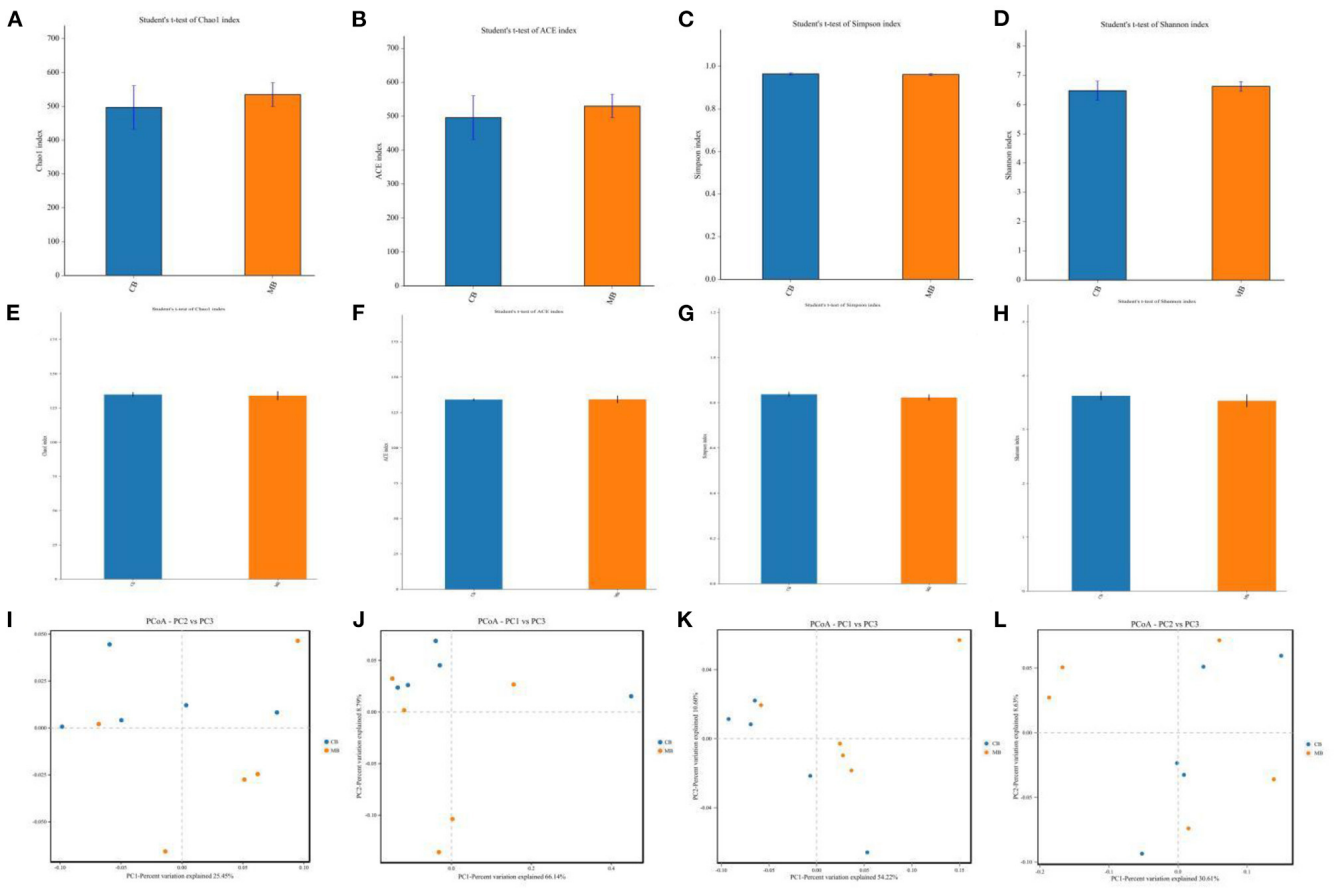
Changes of gut bacterial and fungal diversities associated with mastitis in buffalos. **(A–D)** Represent gut bacterial Chao, ACE, Simpson, and Shannon indices, respectively. **(E–H)** Represent gut fungal Chao, ACE, Simpson, and Shannon indices, respectively. **(I,J)** Represent gut bacterial PCoA maps, whereas **(K,L)** Represent gut fungal PCoA maps.

To further explore the influence of mastitis on the gut microbiota of a buffalo, we calculated the alpha and beta diversity indices that could reflect gut microbial diversity. Bacterial and fungal Good's coverage estimates in each sample of CB and MB were almost 100%, implying excellent coverage. Furthermore, there were no significant differences in the bacterial and fungal Chao1 (496.79 ± 144.06 vs. 534.90 ± 79.22, *P* = 0.62; 134.77 ± 3.85 vs. 133.91 ± 7.35, *P* = 0.82), ACE (495.99 ± 143.39 vs. 529.78 ± 77.53, *P* = 0.65; 134.01 ± 2.01 vs. 134.20 ± 5.87, *P* = 0.94), Simpson (0.96 ± 0.011 vs. 0.96 ± 0.010, *P* =0.69; 0.83 ± 0.025 vs. 0.82 ± 0.030, *P* = 0.44) and Shannon (6.47 ± 0.72 vs. 6.62 ± 0.37, *P* = 0.70; 3.62 ± 0.17 vs. 3.53 ± 0.26, *P* = 0.53) indices between CB and MB (Figures 2A–H). Alpha-diversity analysis indicated that mastitis had no distinct effect on the gut bacterial and fungal diversity and abundance in buffalos. Principal coordinate analysis (PCoA) plots that reflect the similarities and differences among different samples were used to assess the gut bacterial and fungal beta-diversity. Results of the beta-diversity analysis showed that the samples in CB and MB were clustered together, implying similar gut microbial principal components (Figures 2I–L).

### Comparative Analysis of Bacterial Taxonomic Composition

**Figure 3:**
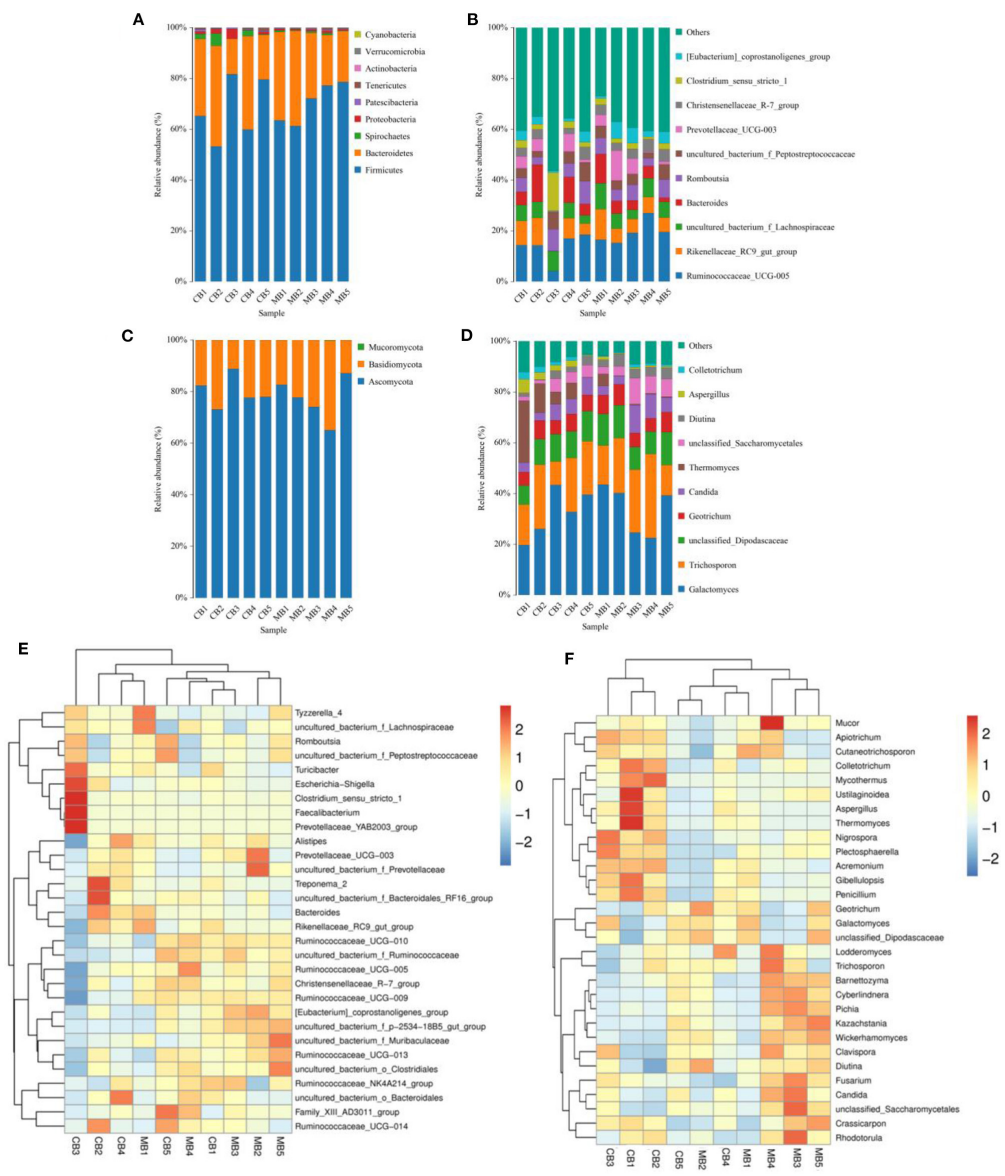
The proportions of preponderant bacterial **(A,B)** and fungal **(C,D)** taxa at the level of phylum and genus associated with mastitis in buffalos. The color-block in the heatmap indicates the normalized relative richness of each bacterial **(E)** and fungal **(F)** genera in healthy and mastitis-affected buffalos.

**Figure 4:**
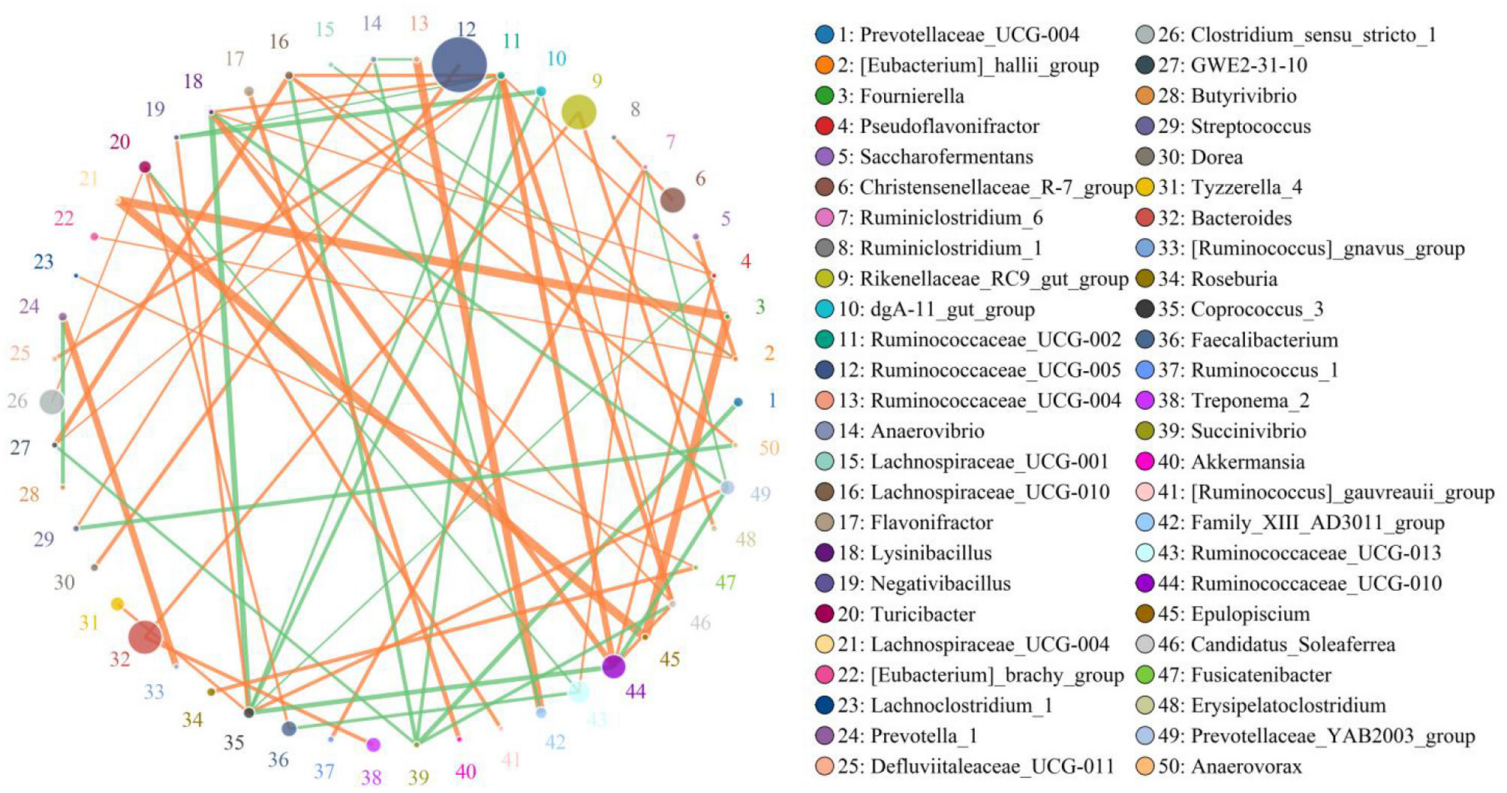
The network diagram visualizes correlations between different bacterial genera. The orange lines indicate a positive correlation and the green lines indicate a negative correlation.

In this microbiome investigation, a total of 9 bacterial phyla and 155 genera were recognized in CB and MB, ranging from 7 to 9 phyla and 103 to 134 genera per sample, respectively. The phyla *Firmicutes* (70.31, 68.19%), *Bacteroidetes* (27.88, 27.50%), *Spirochaetes* (0.53, 1.84%), and *Proteobacteria* (0.56, 1.46%) were the four most preponderant bacterial phyla in samples of CB and MB regardless of health condition, which accounted for approximately 99.00% of the total composition (Figure 3A). Other phyla, such as *Patescibacteria* (0.32, 0.45%), *Tenericutes* (0.25, 0.33%), *Actinobacteria* (0.091, 0.10%), *Verrucomicrobia* (0.028, 0.10%), and *Cyanobacteria* (0.024, 0.020%), in CB and MB were identified in lower abundances. At the genus level, the most dominant bacterial genera in the CB were *Ruminococcaceae_UCG-005* (13.79%) followed by the *Bacteroides* (6.88%) and *Rikenellaceae_RC9_gut_group* (6.42%). However, *Ruminococcaceae_UCG-005* (19.25%), *Rikenellaceae_RC9_gut_group* (7.06%) and *uncultured_bacterium_f_Lachnospiraceae* (6.70%) were abundantly present in the MB (Figure 3B). Bacterial distribution, as well as correlation of both groups during mastitis could also be observed by the clustering heatmap and network diagram, respectively (Figures 3E, 4).

**Figure 5:**
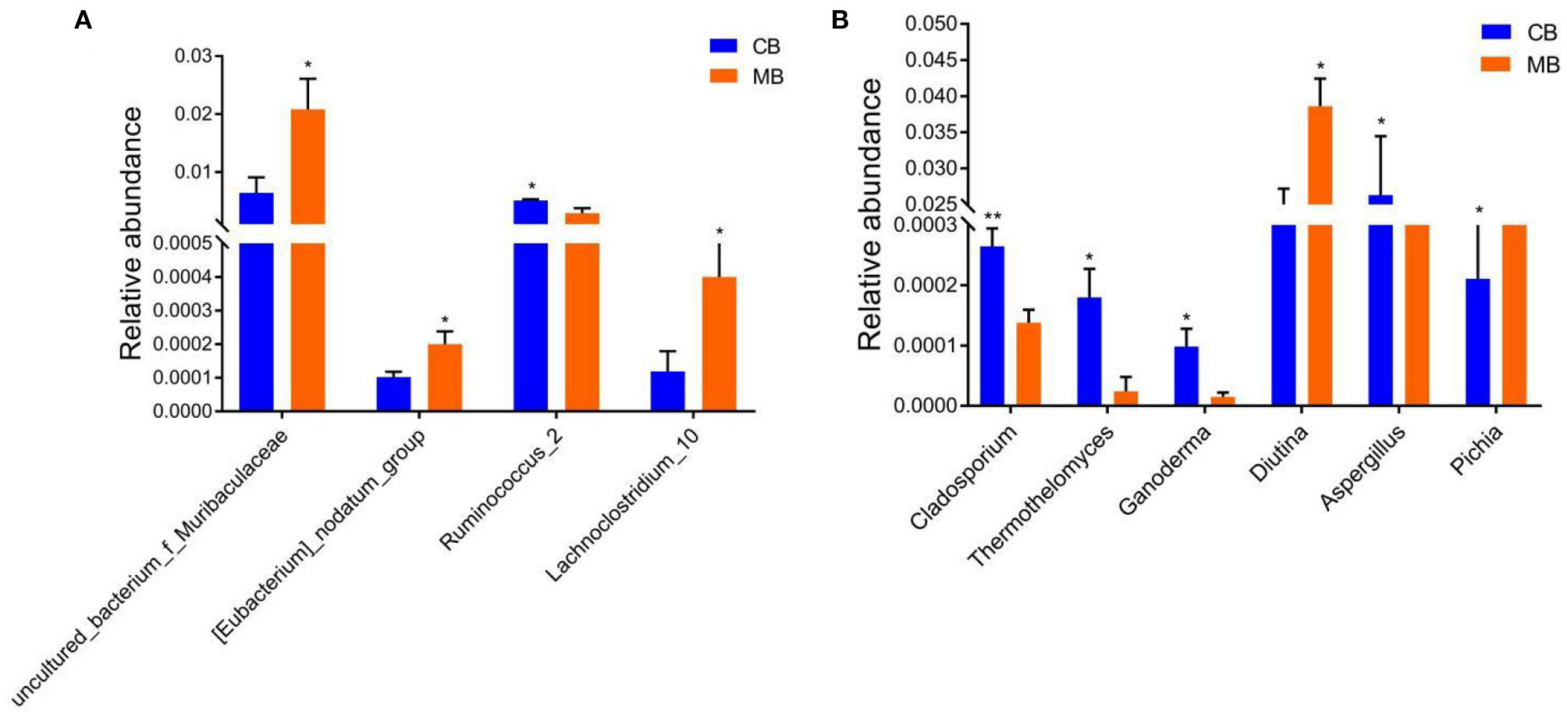
Significant changes in the intestinal bacteria **(A)** and fungi **(B)** associated with mastitis in buffalos. Data was indicated as mean ± SD. *p < 0.05, **p < 0.01.

**Figure 6:**
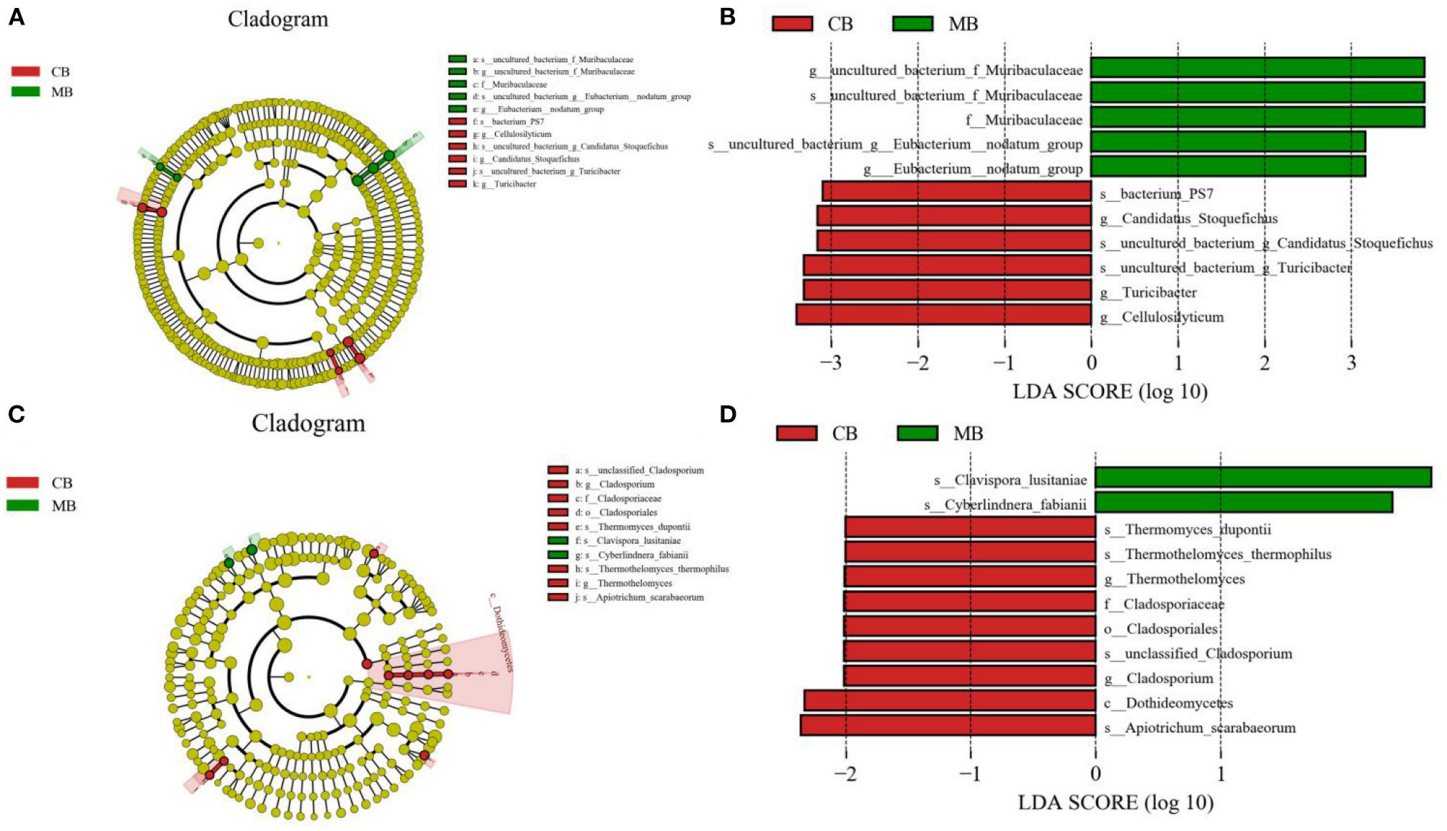
Recognition of differential taxa associated with mastitis in buffalo. Phylogenetic distribution of bacterial **(A)** and fungal **(C)** taxa with obvious differences were visualized through the cladogram. The criterion of bacterial **(B)** and fungal **(D)** significance was performed at LDA scores > 2.

To further assess the influences of mastitis on the gut microbiota in buffalos, we performed Metastats analysis on different classification levels. At the genus level, *uncultured_bacterium_f_Muribaculaceae, Eubacterium_nodatum_group*, and *Lachnoclostridium_10* were significantly more dominant in the MB group than in the CB group, whereas the *Ruminococcus_2* was lower (Figure 5A). Besides the above-mentioned differential taxa, the MB group also showed dramatically lower richness of *Candidatus_Stoquefichus, Turicibacter*, and *Cellulosilyticum* (Figures 6A,B).

### Comparative Analysis of Fungal Taxonomic Composition

There were 3 phyla and 66 genera recognized in the gut fungal community of CB and MB and the dominant phyla and genera were presented in Figures 3C,D. The *Ascomycota* (77.11, 80.05%) and *Basidiomycota* (22.83, 19.91%) were the most prevalent fungal phyla in both groups, making up approximately 99.00% of the overall fungal composition. The *Ascomycota* (77.11, 80.05%) and *Basidiomycota* (22.83, 19.91%) were the most prevalent fungal phyla in both groups, making up approximately 99.00% of the overall fungal composition. At the genus level, the *Galactomyces* (33.66, 32.91%), *Trichosporon* (21.62, 18.51%), and *unclassified_Dipodascaceae* (11.13, 10.24%) were the most prevalent fungal genera in both groups, which accounted for over 60.00% of the total taxonomical group identified. The clustering heatmap also showed the distribution and changes of gut fungal community in buffalo with mastitis (Figure 3F).

Metastats analysis was used to compare the differences in the gut fungal community of both groups (Figure 5B). At the genus level, *Cladosporium, Thermothelomyces, Ganoderma*, and *Aspergillus* were all significantly overrepresented in the CB group, whereas *Pichia* was the most abundant fungus in MB. Similar results were also observed in Figures 6C,D.

## Discussion

Mastitis is a common disease in buffalos that seriously affects milk production and animal health, causing enormous economic loss (23). However, multiple factors, including pathogen infection, unclean environment, nutritional deficiency, and stress reaction, cause mastitis to be difficult to control (23–25). A gut microbial community containing trillions of microorganisms has been demonstrated to be a complex and interactive ecosystem that participates in the positive regulation of host metabolism and health (22, 26). Although, these microorganisms, including bacteria and fungi, colonize the intestine, they can expand their negative impact beyond the gastrointestinal tract and thus cause the development of other diseases (27–29). Recent investigations about gut microbiota have also indicated its key role in the development of mastitis in dairy cows (30, 31). Presently, the study of the relationship between gut microbiota and mastitis has covered many species, but research regarding the gut bacterial and fungal communities in buffalo with mastitis remains scarce. Here, we systematically dissected the gut bacterial and fungal changes in buffalo associated with mastitis and indicated distinct changes in gut bacterial and fungal taxa in buffalo during mastitis.

Growing evidence indicated that the gut microbiota was a dynamic system that was inevitably influenced by multiple intrinsic and extrinsic factors, including diet, age, and sex environment (32–34). Generally, the physiological fluctuations of gut microbiota caused by the above-mentioned factors cannot affect intestinal normal function and homeostasis. However, intestinal-related diseases, such as diarrhea, colitis, and colorectal cancer, have been demonstrated to perturb intestinal homeostasis, resulting in dysbiosis (35, 36). Moreover, recent studies have also shown that diabetes, high blood pressure, and obesity could also cause significant changes in the gut microbiota (37–39). In this study, we selected feces to explore the gut microbial changes in buffalos with mastitis in consideration of the samples' availability and subjects' particularity. Results indicated that there were no significant differences in the gut microbial alpha-diversity between healthy and mastitis-affected buffalo, suggesting that mastitis had no effect on the gut microbial diversity and abundance of the buffalos. Consistent with this study, Ma et al. (3) also demonstrated that there were no obvious differences in the gut microbiota alpha-diversity between healthy and mastitis-affected cows. Notably, although the differences in gut bacterial and fungal diversities between controls and mastitis-affected subjects were not significant, the percentages of some bacteria and fungi altered markedly, implying that these intestinal bacteria and fungi are constantly self-adjusting to the current intestinal environment.

In this microbiome investigation, we observed that Firmicutes and Bacteroidetes were the most preponderant bacteria, whereas Basidiomycota and Ascomycota were the most dominant fungi in buffalos, regardless of health status (40–42). Notably, these microbial phyla have also been demonstrated to be widespread in other ruminants, such as goats, cows, giraffes, and yaks, indicating their key roles in intestinal ecology and function (43, 44). Earlier studies indicated that most members of Firmicutes were intestinal beneficial bacteria involved in the regulation of the immune system, gut microbial homeostasis, and intestinal barrier function (45). Moreover, its members contribute to the degradation of cellulose in ruminants (40). As the dominant bacteria in the gut, Bacteroidetes was responsible for degrading carbohydrates and proteins, showing the great potential for promoting the maturation of the gastrointestinal immune system (43). At the genus level, *Bacteroides* were abundantly present in the healthy buffalo, which was inconsistent with the findings of mastitis-affected buffalo. As intestinal anaerobion, *Bacteroides* can decompose polysaccharides and play a key role in the intestinal ecosystem (46). As intestinal beneficial bacteria, *Rikenellaceae* has been demonstrated to possess multiple probiotic properties and control the development of colitis by regulating T-regulatory cell differentiation (47). *Ruminococcaceae* can degrade cellulose and starch, displaying positive regulation in growing development and feed efficiency (48). Moreover, *Ruminococcaceae*, a potential probiotic candidate, plays active roles in the secretion of short-chain fatty acids (SCFAs), intestinal homeostasis, and host health (49).

The shifts of some specific bacteria and fungi could dissect the potential relationship between gut microbial community and mastitis, thus we further investigated the gut bacterial and fungal changes associated with mastitis. Results showed a significant decrease in 4 bacterial genera (*Ruminococcus_2, Candidatus_Stoquefichus, Turicibacter*, and *Cellulosilyticum)* and 4 fungal genera *(Cladosporium, Thermothelomyces, Ganoderma*, and *Aspergillus*), as well as an increase in 3 bacterial genera (*uncultured_bacterium_f_Muribaculaceae, Eubacterium_nodatum_group* and *Lachnoclostridium_10*) and 1 fungal genus (*Pichia*) in mastitis-affected buffalo. These bacteria and fungi may play an important role in intestinal homeostasis and functions, as well as the development of buffalo with mastitis. *Ruminococcus* and *Cellulosilyticum* have been shown to possess the characteristics of decomposing cellulose and starch (50–52). Notably, *Ruminococcus* is also a potential producer of SCFAs (53). Numerous pieces of evidence indicated that SCFAs play a fundamental role in gut microbial homeostasis and host metabolism (54, 55). Furthermore, SCFAs were also involved in the regulation of intestinal permeability, immunologic function, and cell proliferation (56, 57). Therefore, the higher proportions of *Ruminococcus* and *Cellulosilyticum* in the ruminant are beneficial to maintain energy intake and intestinal function.

Taken together, this study first compared and analyzed the differences in gut microbiota between healthy and mastitis-affected buffalos. Results showed that mastitis did not alter the gut bacterial and fungal diversity, but the proportions of some bacterial and fungal taxa altered significantly. This study also contributes to understanding the gut microbial information of buffalos and shows that the changes in gut bacterial and fungal communities may be an important factor of mastitis. Notably, this research is also beneficial to prevent and treat mastitis in buffalos from the gut microbial perspective.

## Data Availability Statement

The original contributions presented in the study are included in the article/supplementary material, further inquiries can be directed to the corresponding author/s.

## Ethics Statement

The animal study was reviewed and approved by Ethics Committee of the Nanjing Agricultural University. Written informed consent was obtained from the owners for the participation of their animals in this study.

## Author Contributions

XC, MA, and WZ conceived and designed the experiments. MA and WZ contributed sample collection and reagents preparation. HZ, YiW, and XW analyzed the data. XC wrote the manuscript. YuW, MFK, KD, JL, LQ, ZM, and WB revised the manuscript. All authors reviewed the manuscript. All authors contributed to the article and approved the submitted version.

## Funding

This research was financially supported by the National Natural Science Foundation of China (NSFC, Grant Nos. 31872514 and 32172900).

## Conflict of Interest

KD was employed by China Tobacco Henan Industrial Co. Ltd. WB was employed by Nanjing Superbiotech Co. Ltd. The remaining authors declare that the research was conducted in the absence of any commercial or financial relationships that could be construed as a potential conflict of interest.

## Publisher's Note

All claims expressed in this article are solely those of the authors and do not necessarily represent those of their affiliated organizations, or those of the publisher, the editors and the reviewers. Any product that may be evaluated in this article, or claim that may be made by its manufacturer, is not guaranteed or endorsed by the publisher.
